# Modified ribosome profiling reveals high abundance of ribosome protected mRNA fragments derived from 3′ untranslated regions

**DOI:** 10.1093/nar/gku1310

**Published:** 2014-12-29

**Authors:** Teemu P. Miettinen, Mikael Björklund

**Affiliations:** Division of Cell and Developmental Biology, College of Life Sciences, University of Dundee, DD1 5EH Dundee, Scotland, UK

## Abstract

Ribosome profiling identifies ribosome positions on translated mRNAs. A prominent feature of published datasets is the near complete absence of ribosomes in 3′ untranslated regions (3′UTR) although substantial ribosome density can be observed on non-coding RNAs. Here we perform ribosome profiling in cultured *Drosophila* and human cells and show that different features of translation are revealed depending on the nuclease and the digestion conditions used. Most importantly, we observe high abundance of ribosome protected fragments in 3′UTRs of thousands of genes without manipulation of translation termination. Affinity purification of ribosomes indicates that the 3′UTR reads originate from ribosome protected fragments. Association of ribosomes with the 3′UTR may be due to ribosome migration through the stop codon or 3′UTR mRNA binding to ribosomes on the coding sequence. This association depends primarily on the relative length of the 3′UTR and may be related to translational regulation or ribosome recycling, for which the efficiency is known to inversely correlate with 3′UTR length. Together our results indicate that ribosome profiling is highly dependent on digestion conditions and that ribosomes commonly associate with the 3′UTR, which may have a role in translational regulation.

## INTRODUCTION

Translation can be subdivided into initiation, elongation and termination. In addition to the core ribosome, a number of auxiliary proteins interact with ribosomes and mRNA to assist through each phase. Initiation is usually the rate limiting step in the translation of most mRNAs after which translational elongation commences. When ribosome encounters a stop codon, the newly synthesized polypeptide is released and the ribosome subunits dissociate from mRNA. The current understanding is that the action of release factors eRF1 and eRF3 results in the release of the newly synthesized polypeptide. ABCE1 (Rli1 in yeast) then acts to release the 60S subunit of the ribosome leaving the 40S subunit with the deacylated tRNA complex bound to the mRNA (reviewed by (1,2)). The remaining ribosomal complex can then fully dissociate or engage in reinitiation. The mRNAs engaged in active translation are thought to form a loop due to interactions of polyA binding protein with initiation factors (2–4), suggesting that incomplete dissociation and recycling at the stop codon may also potentially facilitate transfer of the ribosome back to the 5′ untranslated regions (5′UTRs) for additional rounds of translation. Apart from a few exceptions, ribosomes have not been observed on 3′UTRs in large quantities. It was recently described that yeast mutant, where translation termination was modified, displayed high levels of ribosomes in the 3′UTRs (5). 80S ribosomes have also been observed on large intergenic non-coding RNAs (lincRNAs) (6,7). One further situation where ribosomes have been observed in 3′UTR is stop codon read-through (8).

Ribosome profiling using high-throughput sequencing is an increasingly popular method for monitoring translational events and it has provided major insights into translational control in bacterial, yeast, zebrafish, fruit fly and mammalian cells (8–12). In this assay, ribosome binding protects mRNA from cleavage by nucleases generating a collection of sequence fragments (13). These can be separated based on their characteristic size (∼30 nt) and sequenced to gain information on the exact positions of ribosomes (ribosome protected footprints, RPFs) on mRNAs using high-throughput sequencing (12). Combining this information with mRNA sequencing may additionally provide an estimate of the translational efficacy for each gene, especially when properly correcting the ribosomal footprint counts for mRNA expression levels (14). Despite the elegance of the ribosome profiling method, interpretation of the sequencing read counts is not without potential problems (15). Although it is possible to separate polysomes from monosomes this may not be sufficient to separate RPFs of ribosomes that are actively translating from those which are not translating, as not all 80S ribosomes are actively engaged in translation (6,16). Comparisons of the ribosome density before and after putative stop codons were needed as an additional measure to identify regions with are translated (6).

It has not been assessed, how nuclease choice affects ribosomal profiling. It is possible that nuclease accessibility to the ribosomes may vary at different phases of translation when ribosomes are bound by a variety of initiation, elongation or termination factors. Apart from two studies (8,17), RNase I digestion has been exclusively used in eukaryotic ribosome profiling. An alternative digestion method uses micrococcal nuclease (MN) (13), which has been used primarily with bacterial ribosome profiling as RNase I binds bacterial ribosomes (18). Here we directly compare RNase I and MN digestions in cultured *Drosophila* and human cells. Although the data is largely concordant, the two digestion methods yield different quantities of RPF reads from specific sets of genes. RNase I digestion produces more RPFs from translation initiation sites. More strikingly, MN digestion yields high abundance of reads on the 3′UTRs. Repeating ribosome profiling under different digestion conditions indicated that the RPFs on 3′UTRs are more sensitive to digestion under typically used conditions. The 3′UTR RPFs were also found when ribosome profiling was performed after affinity purification of ribosomes.

## MATERIALS AND METHODS

### Cell culture, lysate preparation and nuclease digestion

Kc167, U2OS and HeLa cells were cultured in the presence of 10% fetal bovine serum (FBS) as described (19). For ribosome profiling cells were grown to ∼70% confluence and four 75 cm^2^ flasks of U2OS cells and 15 ml of Kc167 cells were used. Both cells were stimulated with 10% extra FBS for 1 h followed by 250-μM cycloheximide for 10 min. Cells were rinsed twice with cold phosphate buffered saline and lysed in 1-ml of our ‘standard’ polysome extraction/lysis buffer (1% deoxycholate, 1% NP40, 10-mM HEPES (pH 7.4), 350-mM KCl, 5-mM MgCl2, 5-mM CaCl_2_, 250-μM cycloheximide, 1x protease inhibitors (Sigma, P8340) and 2-μl RiboLock RNase inhibitor (ThermoFisher)). Lysates were centrifuged for 10 min at 16000 g and supernatant (leaving lowest ∼25% behind) was used for ribosome profiling. For *Drosophila* embryo nuclease digestions 0–4 h old *yw* embryos were collected and lysed in polysome extraction buffer using a Precellys homogenizer (Bertin Technologies). The major differences in our conditions compared to others using MN with eukaryotic cells (8) is that we stimulate translation with additional serum, use higher salt concentration (350-mM KCl) and 0.5-M sucrose cushion (20) and include deoxycholate in lysis buffer.

The lysate samples were split into two different digestion samples and digested with either 200 U of Escherichia coli maltose binding protein (MBP)-RNase I (New England BioLabs) or 100 U of MN (ThermoFisher) by incubating for 40 min (U2OS) or 60 min (Kc167) at RT with slow mixing. Two unitsof DNase I (ThermoFisher) was also added to each U2OS sample at the start of digestion. MN reaction was stopped by the addition of ethylene glycol tetraacetic acid (EGTA) to 10 mM final concentration.

For comparisons of digestion conditions U2OS and HeLa cells were treated with FBS and cycloheximide as before and lysed with 0.7 ml of modified lysis buffer as follows: Control samples (standard buffer), low NaCl sample (100-mM NaCl, no KCl), low KCl sample (100-mM KCl), TritonX-100 sample (0.5% TritonX-100, no deoxycholate or NP40), high MN sample (standard buffer)). In addition a sample without FBS pre-treatment was made using control polysome extraction buffer. After lysis samples were processed as before, with the exception of high MN samples, which was digested with 600 U of MN for 2.5 h followed by 20-mM EGTA treatment.

In addition, a set of U2OS samples was prepared, where cycloheximide was omitted or digestion was performed in the presence of excess mouse RNA. Total RNA from mouse liver was isolated using trizol. Ten times excess mouse RNA (as measured by A_260_) was added to U2OS ribosome profiling sample after cell lysis, but before digestion. The digestion for all of the samples was made using our standard conditions and MN.

### Isolating of the ribosome footprints

The digested samples were centrifuged at 6000 *g* for 5 min to pellet insoluble material. The ribosomes were separated by sucrose step centrifugation using 0.5 M sucrose in polysome extraction buffer supplied with SUPERase-In (1/500) (Ambion). A 0.35 ml lysate was layered on top of 0.2-ml sucrose cushion and centrifuged at 100 000 *g* for 45 min (55 000 rpm using TLA 120.1 rotor). Supernatant was removed and pellet was suspended in 600 μl Qiazol reagent (Qiagen). Samples were incubated for 15 min at 70°C with mixing to dissolve the pellet after which 200 μl of chloroform was added, the samples were vortexed and centrifuged for 10 min at 12 000 *g* at 4°C. RNA was precipitated in the presence of Pink Co-precipitant (Bioline) at −80°C o/n. RNA was washed and suspended in 20 μl of sterile water and mixed with 80 μl 2x Tris-Borate-EDTA (TBE)-urea sample buffer (without Xylene Cyanol FF). Samples were run on a 15% TBE-urea gel, the gel was stained using 1x SYBR Gold (Invitrogen) and roughly 25–35 nt sized RNA was cut out of the gel with the help of 28 and 35 nt oligoribonucleotide size markers. The gel pieces were disrupted and RNA was eluted with 300-mM Sodium Acetate (pH 5.0), 1-mM EDTA and SUPERase-In for 30 min at 65°C. RNA was precipitated, centrifuged and washed with 80% ethanol and then suspended in 5 μl of sterile water. RNA yields were quantified with SYBR Gold using Qubit fluorometer (Invitrogen). All samples had over 1-pmol/μl RNA.

### Ribosome affinity purification

*Drosophila* RpS8, RpL13 and RpL22 open reading frames (ORFs) (21) were transferred to an expression vector containing OpIE2 promoter and which adds O^6^-alkylguanine-DNA-alkyltransferase (AGT)-2xprotein G tag to the C terminus of the ORF. The plasmids were transfected into Kc167 cells using FuGENE HD (Promega) and stable cell lines obtained by selection on 250-μg/ml zeocin for three weeks. The incorporation of the tagged ribosomal proteins to the ribosomal fraction was checked by separating the crude ribosomal fraction using sucrose cushion as described above followed by western blotting with infrared dye conjugated anti-rabbit IgG, which detects the protein G part of the epitope tag. For ribosome profiling, 30 ml of each culture were lysed and digested as before with MN (200 U). Digestion was stopped with EGTA and 350 μl goat anti-rabbit IgG magnetic bead suspension (New England BioLabs) together with SUPERase-In (1/500) were added to the lysates and incubated o/n at 4°C with gentle mixing. The beads were collected and washed 3× with the lysis buffer. RNA was extracted from the beads using 500-μl Qiazol reagent and the ribosome profiling was continued as before.

### Library generation and sequencing

Five to fifteen picomoles of each RNA sample was used for phosphatase and polyA tailing reaction by mixing 3.25 μl of RNA with the following: 0.5 μl 10x polyA buffer (0.5-M Tris-HCl, pH 7.0, 2.5-M NaCl, 100-mM MgCl2, 50-mM dithiothreitol (DTT), 1-mg/ml BSA), 0.25-μl Yeast polyA polymerase (600-U/μl, Affymetrix), 0.5 μl of 10-mM ATP, 0.3-μl (3 U) T4 polynucleotide kinase (ThermoFisher), 0.2-μl SUPERase-In. Samples were incubated at 37°C for 3 h. Note that RNase I digestion results in cyclic 2′3′-phosphate, which will be resolved by the inherent phosphatase activity of the T4 polynucleotide kinase. Four microliter of each sample was mixed with 1 μl of 10-μM abasic-oligo dT primers containing random 8-nt sequence used for absolute molecular counting (19,22,23), incubated at 72°C for 3 min and put on ice. Reverse transcription was started by adding the following to the samples: 5.25-μl H_2_O, 3-μl 5x RT-buffer (ThermoFisher), 0.75 μl of 10-mM dNTP, 0.25-μl SUPERase-In and 0.4-μl Maxima Reverse transcriptase (75-U, ThermoFisher). Samples were incubated at 50°C for 1 h, heat inactivated at 85°C for 6 min after which 0.5-μl RNase H (ThermoFisher) was added and samples were incubated at 37°C for 30 min. Reverse transcription products were purified on 10% TBE-urea gel and cDNA was eluted into 700 μl of DNA gel elution buffer (1x TE + 300-mM NaCl) by incubating o/n at 4°C with mixing on Eppendorf Thermomixer. cDNA was precipitated as before, samples were centrifuged for 10 min at 16 000 *g*, pellets were washed and cDNA was suspended into 5 μl of circularization mix (0.5-μl CircLigase buffer (10x), 0.25 μl of 50-mM MnCl_2_, 0.25-μl CircLigase II (Epicentre), 1 μl of 5 M betaine, 3-μl sterile water). Samples were incubated o/n at 60°C and heat-inactivate for 10 min at 80°C. cDNA was linearized by adding 3 μl of relinearization supplement (50-mM KCl, 1-mM DTT) followed by 0.7-μl (7 U) APE1 (New England Biolabs). Samples were incubated for 1 h at 37°C. Libraries were then amplified using 18 cycles of polymerase chain reaction (PCR) with Solexa-primer-F and R and Phusion DNA polymerase (ThermoFisher). PCR products were separated on agarose gel, eluted using Qiagen MinElute Gel extraction kit and quantified with Quant-it PicoGreen DNA dye (LifeTechnologies) using Qubit fluorometer (LifeTechnologies). Samples were sequenced using Illumina GA_iix_ and HiSeq using single end sequencing with 50-bp read length at the GenePool sequencing facility (University of Edinburgh).

### Primers used

#### Abasic-oligo dT primers

The following barcoded oligonucleotides were used to allow multiplexing of sequencing:

RiboProf_umiXXX
/5phos/XXXNNNNANNNNAGATCGGAAGAGCGTCGTGTAGGGA/idSp/CAAGCAGAAGACGGCATACGAGCTCTTCCGATCTTTTTTTTTTTTTTTTTTVN, where XXX denotes the three nucleotide barcode.

#### Library amplification primers

Solexa-primer-F AATGATACGGCGACCACCGAGATCTACACTCTTTCCCTACACGACGCTCTTCCGATCT

Solexa-primer-R CAAGCAGAAGACGGCATACGAGCTCTTCCGATC

### Proteomic analysis and nuclease ribosome interactions

Cell culture samples for mass spectroscopy were collected, digested and centrifuged through sucrose cushion as for ribosome profiling. The crude ribosomal pellet was washed with sterile water and suspended in to 10-mM Tris–HCl, 150-mM NaCl, 1-mM EDTA using a Precellys homogenizer. Sample proteins were separated on polyacrylamide gel electrophoresis (PAGE) and analysed in five separate samples using Velos Orbitrap mass spectrometer (ThermoFisher). The relative protein quantification was done using exponentially modified protein abundance index scoring (emPAI) (24) obtained from Mascot database search engine. Western blot validations of proteomics data were done from U2OS cells collected as for ribosome profiling, but digested with indicated amounts of nucleases. Western blots are from ribosomal pellets following sucrose cushion centrifugation.

For nuclease ribosome interaction studies U2OS cells were collected, digested and centrifuged through sucrose cushion as for ribosome profiling. Ribosomal pellet was suspended in to polysome extraction buffer using sonication. The presence of indicated proteins was detected using western blot. RNase I was a fusion with MBP and 8 μg of MBP was added to digestions as a control.

The following antibodies were used: anti-MBP (New England BioLabs, E8032S), anti-GAPDH (CST, #5174), anti-MN (Acris Antibodies, AP21415AF-N), anti-RpS6 (CST, #2317), anti-RpS3 (CST, #9538), anti-RpL13a (CST, #2765), anti-RpL9 (Abcam, ab50384) and anti-RpS7 (Abcam, ab57637). Antibodies were detected as in (19).

### Polysome analysis

Sucrose gradient analysis was performed using HeLa cells as a source of human ribosomes. Cells were treated with FBS and collected as for ribosome profiling, but using polysome extraction buffer with 1% Triton X-100 instead of NP40 as NP40 causes significant background. Cell lysate was divided into equal portions for different digestions. Control samples were treated with 30-U/ml SUPERase-In instead of nucleases. 10-ml sucrose density gradients (10–50% w/v) were prepared in buffer containing 20-mM Tris–HCl (pH 7.5), 140-mM KCl, 5-mM MgCl_2_, 30-U/ml SUPERase-In. Samples were centrifuged 2 h, 38 000 rpm, at 4°C using SW 41 Ti rotor (Beckman Coulter) and gradients were analysed using an TELEDYNE Isco gradient fractionation system and PeakTrak software with continuous A_254_ monitoring.

### Bioinformatic analysis

The sequencing reads were assigned for each sample using their respective barcode and mapped to human and *Drosophila* transcriptome (ENSEMBL version 73) by using the cDNA sequences encoding for the longest open reading frame for each gene as a reference sequence. Mapping was done using the program Bowtie. Custom shell scripts and R was used for further analysis. Briefly, short reads (<20 nt) and sequences containing 16 or more successive A nucleotides were removed from analysis. Reads mapping to non-coding RNAs were first removed and remaining reads were then mapped against the coding cDNAs. Only reads mapping uniquely to the coding cDNAs were accepted for analysis. We used 3′UTR annotations as such without extending them, although this may result in cases where ribosomes that overlap with polyA sequences are missed (e.g. Figure 4d). Unique reads were identified by collapsing duplicate reads with same molecular barcode (19,22,23). Ribosome profiling data was compared to U2OS cell proteomes (25,26) by mapping the protein IDs to ENSG IDs. Ribosomal profiling counts were normalized to reads per kilobase of cDNA for this analysis. For comparison of data from *Drosophila* S2 cells treated with MN (8), deposited sequences from sucrose cushion isolated ribosomes from S2 cells lysed in buffer containing 150-mM Na^+^, 5-mM Mg^2+^ were downloaded from NCBI short read archive (http://www.ncbi.nlm.nih.gov/sra, accession number SRX327694, run SRR942878). These sequences were mapped after trimming the adapter sequence and analysed as *Drosophila* Kc167 samples.

**Figure 1.**
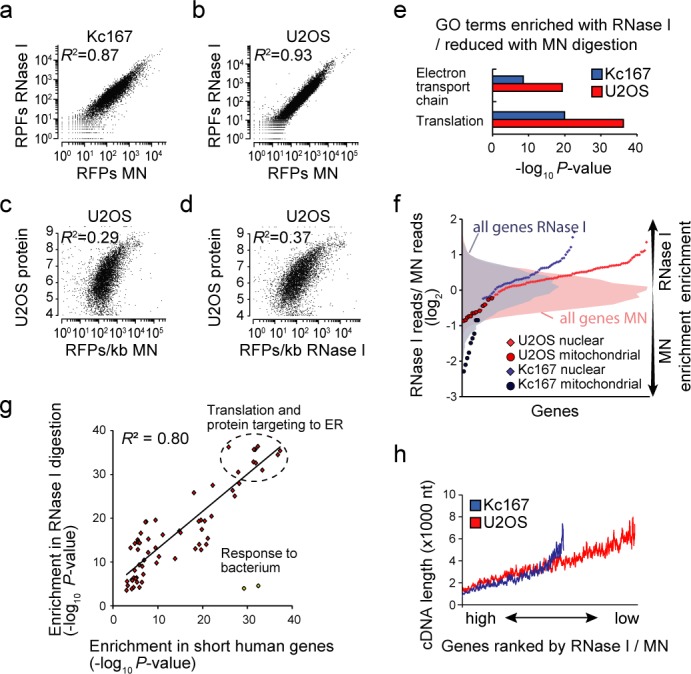
RNase I enriches short nuclear genes in comparison to MN. (**a**) Correlation of ribosome protected footprint (RPF) counts between the two digestion methods in Kc167 cells. (**b**) Correlation of RPF counts between the two digestion methods in U2OS cells. (**c**) Correlation between MN digested U2OS RPF counts (normalized to cDNA length) and U2OS protein levels (26). (**d**) Correlation between RNase I digested U2OS RPF counts and U2OS protein levels. (**e**) Gene ontology (GO) analysis of all the genes ranked by their enrichment with RNase I in comparison to MN. Only two GO terms, translation and electron transport chain, were enriched in both cell lines. (**f**) Analysis of individual genes related to the GO term ‘electron transport chain’. Nuclear encoded mRNAs translated by cytosolic ribosomes show enrichment with RNase I digestion, but mitochondrial mRNAs translated by mitochondrial ribosomes enrich with MN. Histograms of the enrichment distribution of all genes are shown on the background to highlight the levels of enrichment. (**g**) *P*-value correlation between GO terms enriched with RNase I and GO terms enriched based on gene length in U2OS cells. Longest coding transcripts of all human genes were used to analyse for enrichment in shorter genes. The two outlier GO terms related to ‘response to bacteria’ response were omitted from the calculation of Pearson correlation (*R*^2^). (**h**) Comparison of cDNA length (sliding window of 50 genes) with RNase I enrichment in both Kc167 and U2OS cells. Pearson correlation value (*R*^2^) is indicated in panels (a–dand g). All GO term analyses were done based on all RPFs on cDNA.

**Figure 2.**
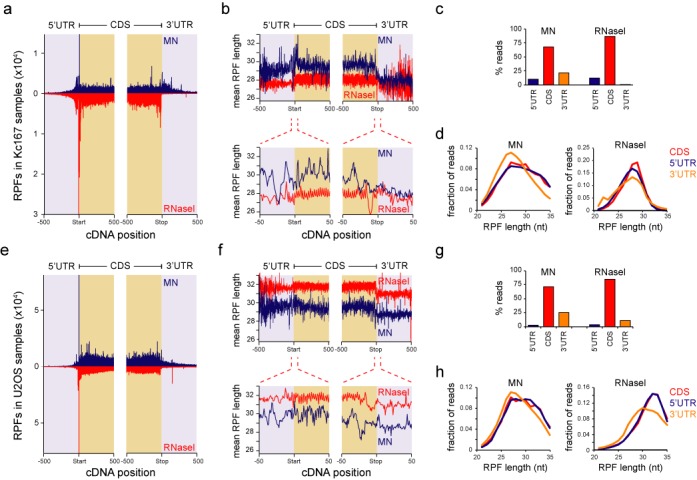
MN digestion results in high abundance of shorter RPFs on 3′UTRs. (**a**) RPF counts from all genes in a 1000 nucleotide region around start and stop codons in Kc167 cells. MN counts are shown in blue (above zero) and RNase I counts in red (below zero). Light blue background depicts UTRs and light brown depicts CDS. (**b**) Mean RPF length at each position relative to start and stop codon in Kc167 samples (top panel). MN counts are shown in blue and RNase I counts in red. Note the drop in the mean RPF length at the stop codon. 10x zoom-in is displayed at the bottom panel. The 3-nt pattern in the RPF lengths can be seen within the CDS with RNase I digestion, but this pattern is disturbed on the UTRs. (**c**) Relative RPF amounts on different mRNA regions with MN (left) and RNase I (right) digestions in Kc167 cells. (**d**) RPF length distributions on different mRNA regions with MN (left) and RNase I (right) digestion in Kc167 cells. (**e**) Same as (a), but data from U2OS cells. (**f**) Same as (b), but data from U2OS cells. (**g**) Same as (c), but data from U2OS cells. (**h**) Same as (d), but data from U2OS cells. Note that all RPF locations match to the start of the read, not the E, P or A sites of the ribosome.

**Figure 3.**
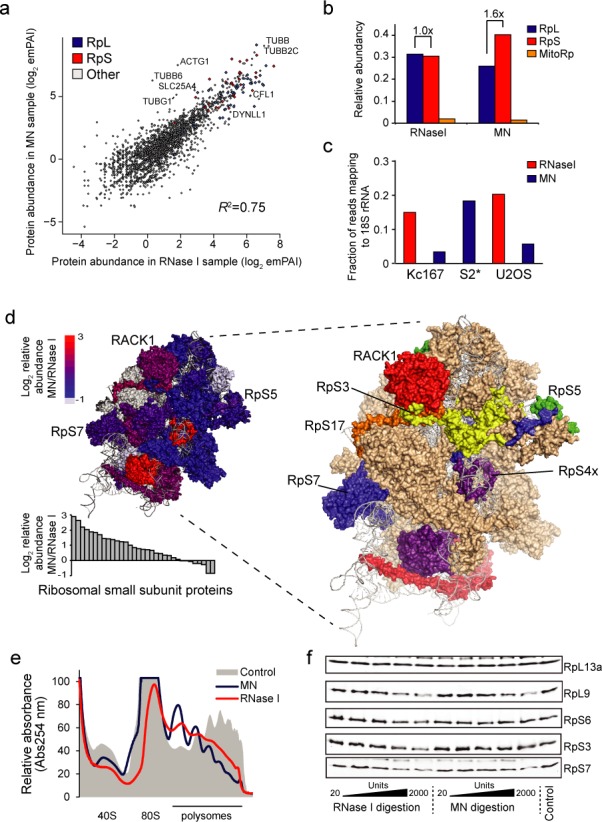
RNase I digests small ribosomal subunit. U2OS cells were digested with MN and RNase I as for ribosome profiling and protein levels were analysed by mass spectrometry after ribosome separation using sucrose cushion centrifugation. (**a**) Correlation of protein abundances (log_2_ of emPAI scores) between the two digestions. Ribosomal proteins and Pearson correlation value (*R*^2^) are indicated. (**b**) Average protein abundances (normalized emPAI scores) for small subunit (RpS), large subunit (RpL) and mitochondrial (MitoRp) ribosomal proteins with MN and RNase I digestions. Relative enrichments of RpS compared to RpL proteins with RNase I and MN are indicated, suggesting differences in ribosome degradation. (**c**) Fraction of ribosome profiling sequencing reads mapping to 18S rRNA. S2* indicates Drosophila S2 cell sample from (8). (**d**) Visualization of the relative changes in RpS proteins based on the mass spec data. Upper left structure shows log_2_ relative abundance of RpS proteins (MN/RNase I) mapped on human 40S ribosome (PDB accessions 3J3D (18S rRNA) and 3J3A (RpS proteins)). Grey proteins were not identified by mass spectrometry. Structure on the right, shows the most MN-enriched proteins (log_2_ fold change > 1.5) colour coded individually. Other proteins are in light brown and rRNA is in grey. The views are from the solvent side of the 40S ribosome. Bar chart on the bottom left shows the relative enrichment of individual RpS proteins. (**e**) Sucrose gradient analysis of MN (left) and RNase I (right) digested HeLa cells. Undigested lysate is shown as a control. Equal amount of cell lysate was used for each sample. (**f**) Western blots of U2OS cell lysates digested with indicated amounts of nucleases and centrifuged through sucrose cushion. Some ribosomal proteins, like RpS3, are dissociated from ribosomes more with RNase I than with MN. See also panel (d). Nuclease digestions were carried out under same conditions as for the ribosome profiling samples.

**Figure 4.**
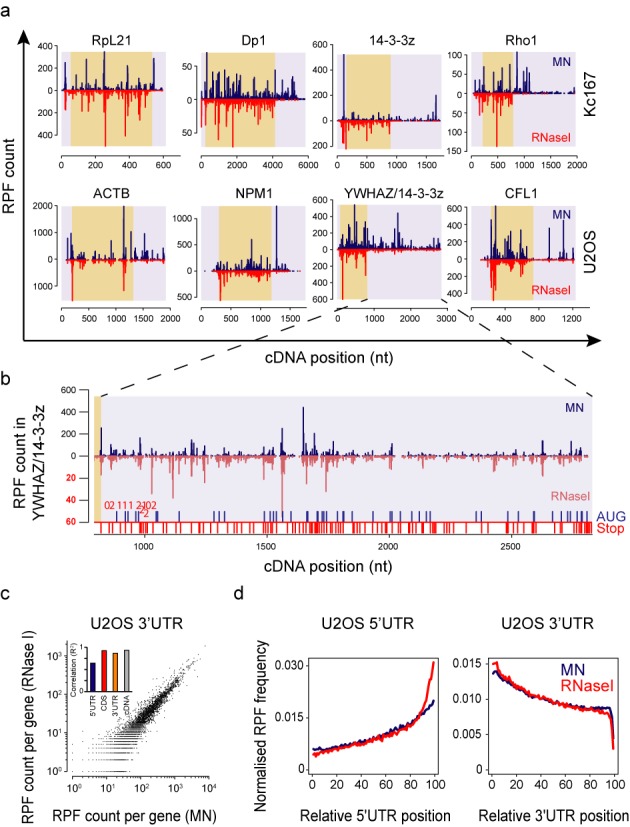
RPFs on 3′UTRs originate from same positions but in different quantities. (**a**) Examples of *Drosophila* (top row) and human genes (bottom row) with abundant RPFs on 3′UTR. MN samples are in dark blue and RNase I samples in red. For colour coding, see Figure 2. Note the common locations of many large RPF peaks on CDS. (**b**) Detailed view of the human YWHAZ/14–3–3z gene 3′UTR from panel (a). The RNase I RPFs (light red, below zero) are plotted in 10x magnification to better visualize RPF peak locations (magnified scale in red). Stop codons and AUG codons are indicated immediately above the x-axis with red and blue ticks, respectively. Red numbers above the tick marks indicate the reading frame relative to CDS for most proximal stop codons (0 = in frame with CDS, 1 = +1 and 2 = +2 relative to reading frame). (**c**) Correlation of RPF counts on 3′UTRs between MN and RNase I digestions in U2OS cells. Inset displays the Pearson correlation values (*R*^2^) for RPFs on each individual cDNA region. (**d**) Normalized RPF density in U2OS samples along 5′UTR and 3′UTR relative to UTR lengths rather than absolute position as in Figure 2. MN data are shown in blue and RNase I data in red. Note the RPF density increase towards the start codon in the 5′UTR data, especially with RNase I, and the continuous drop in 3′UTR RPF density. The final 3′UTR density drop in the extreme 3′ end is due to mapping as we do not extend the 3′UTR sequence.

**Figure 5.**
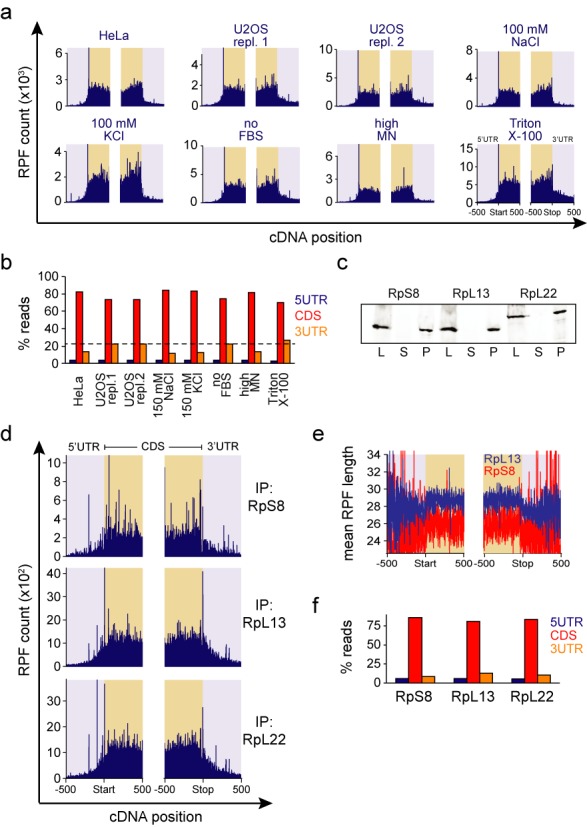
3′UTR RPFs associate with ribosomes under multiple conditions. (**a**) RPF counts of all genes in a 1000 nucleotide region around start and stop codons from U2OS cells digested with MN under different conditions (see ‘Materials and Methods’ section for details). Control conditions, which were also used for HeLa cells, are the same as in Figure 2. (**b**) Quantification of relative RPF amounts on different mRNA regions for the samples shown in panel (a). The dotted line illustrates the mean 3′UTR RPF abundance in control samples. (**c**) Western blots of Kc167 cells transfected with tagged ribosomal proteins. Cells were lysed and centrifuged through sucrose cushion to compare the accumulation of the tagged ribosomal protein in different fractions. L = lysate, S = supernatant, P = pellet (ribosomal fraction). (**d**) RPF counts of all genes from immunoprecipitated (IP) ribosomal proteins of Kc167 cells digested with MN. The visualization is as in panel (a). (**e**) Mean RPF lengths for each position relative to start and stop codon for all genes in immunoprecipitated Kc167 samples. RpL13 and RpS8 samples are in blue and red, respectively. Note the drop in mean RPF length at the stop codon. (**f**) Relative RPF amounts on different mRNA regions after ribosome immunoprecipitation of Kc167 cells.

The reads on 5′UTR were defined as those whose start was located more than 12 nt upstream from AUG codon and for 3′UTR those with more than 18 nt upstream stop codon to reflect approximate positioning of ribosomes on start and stop codons (12). Data was plotted using Microsoft Excel or in R environment. For analysis of feature correlations (e.g. Figure 5b and c), we ignored genes with <100 counts. The high salt concentration used for lysis and digestion precludes reading frame analysis on the coding sequence as shown previously (8).

For metagene analysis, RPF counts were summed for each position (e.g. Figure 2) or relative positions along UTRs were calculated by dividing the sequence position relative to start (in the case of 5′UTR reads) and stop (in the case of 3′UTR reads) with UTR length (e.g. Figure 4d). The footprint counts at each relative positiion in RNase I and MN samples were normalized to total read counts in UTR to simplify comparison between nucleases in Figure 4d.

Gene ontology (GO) analysis was performed using KEGG pathway enrichment function within STRING database (27) and by using GOrilla software, which analyses enrichment from a ranked list of genes (28). GC content was analysed for the complete sequences obtained from ENSEMBL. For the analysis of local GC content, sequences of RPF length starting from the first RPF position were retrieved along with identical length sequence immediately upstream and downstream of the RPF and plotted as a boxplot in R. Motif analysis was performed with meme (29) (version 4.4.0) using a second order background model generated from human 3′UTR sequences containing 17-nt CDS flank and using the following options *-mod zoops -nmotifs 6 -minw 6 -maxw 10 -evt 0.01* to analyse a 30-nt sequence from all 3′UTR peak positions with more than 30 reads and which came from the Top100 genes with most reads on 3′UTR in U2OS samples treated with MN. The same Top100 genes were also used to identify known RNA binding motifs and predicted miRNA binding sites (30,31). Background was estimated using five randomly generated sequence sets of the same size and with same nucleotide frequencies. Individual RNA binding motifs and miRNA binding sites were considered to be enriched if the 3′UTR dataset had more sites than the mean and three standard deviations in the random sets.

### Ribosomal protein mapping to ribosome structure

40S human ribosome structure (PDB accessions 3J3D (18S rRNA) and 3J3A (RpS proteins) (32)) were downloaded and visualized with MacPyMOL (DeLano Scientific). Log2 fold change of enrichment of RpS proteins was calculated based on mass spectrometry data and assigned with colour-coding. Surface representations were used for proteins.

### Statistical analysis

The GC content of RPFs on 3′UTR was compared to the GC content to those of 30-nt sequences immediately before and after the footprint for all footprints identified on 3′UTRs. Statistical signficance was analysed by ANOVA with Tukey's post hoc test using *aov* and *TukeyHSD* functions in R environment.

## RESULTS

### Comparative analysis of ribosome profiling with RNase I and MN

We performed ribosome profiling in *Drosophila* embryonic Kc167 cells and human U2OS osteosarcoma cells using RNase I and MN digestions under conditions aimed to minimize any other effects than those due to nuclease choice. We stimulated translation with additional 10% FBS for 1 h before lysis and the samples for RNase I and MN digestions were derived from the same cell lysates and same digestion conditions were used for both nucleases. We used the original polyA tailing protocol as this was shown to be superior to the ligation-based approach in terms of evenness of RPF coverage (12). We also included random oligonucleotide sequences in adapter primers to allow absolute molecule counting in order to reduce PCR-based bias. Finally, we did not attempt ribosomal RNA (rRNA) removal during sample preparation and used high salt digestion conditions to minimize weak ionic protein–mRNA interactions, which could lead to false positive footprints derived from other RNA binding proteins (RBPs).

Library generation from U2OS cells was robust. However, we experienced some difficulties in preparing high-coverage ribosome profiling samples from *Drosophila* cells as these libraries had high levels of rRNA contamination. This could be traced to the peculiarities in the *Drosophila* ribosome structure. Unlike in other common eukaryotes, the *Drosophila* 5.8S rRNA subunit is formed from two separate rRNAs, the 2S and the mature 5.8S rRNA (33). Comparison of the RNA digestion patterns from Kc167 cells using PAGE indicated a prominent band of 30 nt in both MN and RNase I digested samples, which we identified as 2S rRNA (Supplementary Figure S1a). MN digestion did not yield any other major bands, which could interfere with footprint analysis. Two additional distinct bands were visible in RNase I treated Kc167 samples. These nuclease digestion patterns were highly reproducible and typical for *Drosophila* RNA as digestion of 0–4 h old *Drosophila* embryos resulted in an identical pattern (Supplementary Figure S1a). Thus MN digestion is better suited for *Drosophila* ribosome profiling as also noted previously (8), but nevertheless *Drosophila* samples suffer from high 2S rRNA contamination as the 30-nt long 2S rRNA co-migrates with the RPFs.

Comparison of the total RPF counts on cDNA of each gene for MN and RNase I-digested samples displayed Pearson correlations of *R*^2^ = 0.87 and *R*^2^ = 0.93 in *Drosophila* and human cells, respectively (Figure 1a and b; all data in Supplementary Tables S1 and S2). Although the overall correlations were similar in both cell types, the reproducibility of ribosome profiling has been shown to be much better even between biological replicates (up to *R*^2^ = 0.998 in (8)), and we also routinely obtain *R*^2^ > 0.96 for biological replicates (Supplementary Figure S1b). This lower correlation between digestions suggests that the digestion method may influence data interpretation. Comparison of U2OS RPF counts on cDNA with protein levels from U2OS cells using two independent mass spectrometry datasets (25,26) displayed a slightly better correlation with RNase I (*R*^2^ = 0.37 – 0.43) than with MN digestion (*R*^2^ = 0.29 – 0.35) (Figure 1c and d; Supplementary Figure S1c and d). The U2OS ribosome profiling correlations with protein levels are as good as those observed in yeast (12).

### Different gene sets are enriched depending on digestion method

The unexpectedly low concordance between nucleases suggests that the differences between these nucleases are non-random. We analysed if any gene sets are enriched between RNase I and MN digestion. Genes were ranked by the ratio of RPFs on cDNA between the two digestions and then analysed for gene set enrichment using GO. In both cell lines, ribosomal protein encoding genes and electron transport chain associated genes were enriched with RNase I digestion relative to MN (Figure 1e and Supplementary Figure S1e and f). No other functional groups were enriched with RNase I digestion in both organisms, while MN digestion enriched only broad GO term categories, such as ‘regulation of cellular process’ (Supplementary Figure S1g). More detailed analysis of electron transport chain genes indicated that most nuclear encoded genes, which are translated in cytosol, were enriched in RNase I samples, whereas mitochondrially translated genes displayed the opposite pattern of enrichment (Figure 1f). This data suggests that the accessibility of RNase I and MN to mRNAs protected by mitochondrial and cytoplasmic ribosomes is different.

The mRNAs encoding for ribosomal proteins and proteins of electron transport chain are highly expressed. We considered the possibility that RNase I digestion produces bias towards more abundant mRNAs. This was not the case as the distribution of reads per gene was similar and MN digestion actually resulted in slightly higher median in both organisms (Supplementary Figure S1h). In addition to being highly expressed, ribosomal protein and electron transport chain protein encoding genes are generally very short. GO term analysis of all human and *Drosophila* genes ranked by their length displays a strong enrichment for the same components enriched with RNase I digestion (Figure 1g and Supplementary Figure S1i). Indeed, we found that the RNase I enrichment correlated over the whole range of cDNA lengths, shorter cDNAs being the most enriched (Figure 1h). Consistent with our data, a higher translation rate of short genes has been observed with ribosome profiling using RNase I digestion (12), although we cannot exclude the possibility that MN underestimates translatability of shorter genes. Overall this data suggests that the nuclease affects the quantitative analysis of the translatability of mRNAs.

### MN digestion yields high amounts of RPFs localizing to 3′UTR

To better understand the differences between the two digestion methods, we performed ‘metagene’ analysis with our ribosome profiling data. As expected, most RPFs were found in coding regions in both species and with both nucleases (Figure 2a and e; Supplementary Tables S1 and S2). Note that the peak positions indicate the beginning of the RPF as MN samples do not allow unambiguous detection of ribosome E, P and A sites from sequencing reads due to broader RPF length distribution (see below and (8)). The RPFs at the beginning of the coding sequences (CDS) were more prominent in RNase I digested samples and MN samples did not display the ribosomal ‘ramp’ after the start codon, as observed with RNase I (12). Unexpectedly, under the conditions used, MN digestions generated up to 20–25% of all reads in the 3′UTR in both Kc167 and U2OS cells, while RNase I sample had very low 3′UTR read counts (Figure 2c and g).

MN digested RPFs were slightly longer than RNase I digested reads in Kc167 cells, but shorter in U2OS cells (Figure 2b and f). This is most likely due to the differences in ribosomes between these species and subsequently the accessibility of nucleases to the ribosome protected mRNA. Interestingly, RPF length varied depending on the location at the cDNA. The mean RPF length at the 5′UTRs was highly variable and this variability increased with distance from the initiation codon, as the read count declined (Figure 2b and f). The RPFs at the 3′UTR were shorter than on the CDS (Figure 2b, d, f and h), and due to larger read counts the variability remained stable, except for Kc167 RNase I sample where read counts were low. RPF length distributions were broader with MN than with RNase I (Figure 2d and h) as also shown before (8). Dunn *et al.*, who also observed 3′UTR ribosomes after MN digestion and ribosome profiling, claimed no difference in median read lengths on 5′UTRs but no data was shown for 3′UTRs (8). We thus analysed their MN-digested *Drosophila* S2 cell data for 3′UTR read lengths. Their S2 cell data displayed qualitatively similar changes in mean RPF lengths and length variation (Supplementary Figure S2a). The abrupt and consistent drop in the mean 3′UTR RPF length suggests that if these 3′UTR footprints indeed derive from ribosomes, they have a different composition or conformation.

A reanalysis of the GO term enrichment on UTRs and CDS between the two digestion methods revealed that translation associated mRNAs are highly enriched with RNase I digestion on CDSs in both organisms (Supplementary Figure S2b). In contrast, 5′UTRs displayed enrichment with MN digestion. The GO term enrichments on CDS were not due to digestion bias of MN based on an analysis of GC content on the coding sequence (Supplementary Figure S2c).

### Analysis of possible alternative RPF sources

It is generally assumed that the reads mapping to mRNAs in ribosome profiling experiments are fragments protected by the mRNA tunnel in ribosomes (12). RBPs are abundant in 3′UTR sequences (34) and some of the 3′UTR reads could derive from RBPs. Fortunately, such interactions of sequence-specific RBPs can be identified through motif searches (35). We selected the Top100 genes with most 3′UTR reads in U2OS sample treated with MN and took all 3′UTR located positions where more than 30 reads are found, resulting in 2174 sequence positions. We searched these sequences for over-representation of motifs and identified that individual motifs are present only in a small fraction of peaks (up to 6% of the 2174 searched positions; Supplementary Figure S3a). More than 80% of the most abundant 3′UTR reads can neither be explained by known RNA binding motifs (30) or predicted microRNA (miRNA) binding sites (31) (Supplementary Figure S3b and c). Altogether, this data suggests that most 3′UTR reads are not derived from sequence motif-dependent interactions with RBPs or miRNAs.

We next performed mass spectrometry analysis from sucrose cushion-purified ribosomal extracts from U2OS cells digested with RNase I and MN in our high salt conditions. The protein levels in these samples correlated well based on comparison of semiquantitative protein abundances (*R*^2^ = 0.75; Figure 3a). Our analysis did not indicate major differences in most abundant RBPs between MN and RNase I digestions. We identified a total of 3775 proteins of which 2366 proteins were observed in both samples (Supplementary Table S3). However, most of these proteins had low abundance and ribosomal proteins constituted for more than a quarter of the total protein (Supplementary Figure S3d). Other abundant protein groups were cytoskeletal proteins, which may reflect the close interactions of cytoskeleton and microtubules with ribosomes to organize localized translation (36). Translation termination related proteins (including eRF1 (ETF1), Dom34-like PELO, eRF3b (GSPT2) and HBS1L) constituted only 0.1% of the total protein in both samples (Supplementary Figure S3b). Together with the motif analysis, these data suggest that 3′UTR RPFs do not originate from non-ribosomal proteins.

### RNase I activity results in partial disintegration of 80S ribosomes

We observed that ribosomal proteins from the 40S (small) subunit (RpS proteins) were more abundant in MN sample compared to RNase I sample (Figure 3b and d). This prompted us to examine if RNase I could cleave 18S rRNA in the 40S subunit of the ribosome and lead to loss of certain RpS proteins. We re-analysed the ribosome profiling sequencing data by looking how frequently MN and RNase I cleave 18S rRNA. Under our conditions, RNase I digestion resulted in much higher percentage of reads mapping to 18S rRNA than MN digestion in both cell lines (Figure 3c), as expected based on PAGE analysis in Kc167 cells (Supplementary Figure S1a). Under the conditions used in (8), and where 3′UTR RPFs were not abundant, MN also resulted in a high level of 18S rRNA cleavage in *Drosophila* S2 cells. The true level of 18S rRNA cleavage may be even higher as an rRNA removal step is included in their protocol. We account the differences between MN digestion in our data and that of Dunn *et al*. to differences in digestion conditions. Digestion patterns in 18S rRNA indicated that while MN and RNase I shared many cleavage sites, there were also unique ones (Supplementary Figure S3e). Note that this analysis only includes cleavages which result in ∼30 nt fragments due to the size selection step in ribosome profiling protocol.

Many of the RpS proteins lost with RNase I digestion are found in the head region of the ribosome although some of these proteins have extensions that expand to other regions of the 40S ribosome (Figure 3d). RNase I was also present in the ribosome pellet fraction after sucrose cushion centrifugation suggesting that RNase I interacts more tightly with rRNA compared to MN (Supplementary Figure S3f). To confirm that RNase I digests ribosomes more than MN, we performed sucrose gradient analysis of samples digested in our high salt conditions. We observed reduced level of especially 80S ribosomes and poorly separating polysomes with RNase I, but not with MN, suggesting that ribosome structure is severely compromised after RNase I digestion (Figure 3e). Furthermore, we performed western blots of ribosomal proteins that were pulled down through the sucrose cushion after digested with different amounts of nucleases. Some ribosomal proteins, like RpL13a, were not affected by digestion while others, like RpS3, which had reduced abundance in the mass spectrometry data, were partly detached with RNase I digestion. Overall, these data indicate that RNase I digestion leads to more cleavage of rRNA and loss of RpS proteins in comparison to MN in our conditions (Figure 3f). We have not found conditions, where we could substantially reduce rRNA cleavage by RNase I. We cannot conclusively distinguish if particular ribosome complexes (for example translating versus non-translating) are more susceptible to RNase I digestion, although this could be a possible explanation for the presence of 3′UTR RPFs in MN samples.

### RPFs on 3′UTR derive from same locations, but in different quantities depending on nuclease

We found thousands of genes with high 3′UTR ribosome occupancy (1600 and 3784 genes with >25% of reads mapping to cDNA in Kc167 and U2OS cells, respectively) using MN (Supplementary Figure S4a and b). A striking observation was that the locations of the larger peaks were often very similar in both MN and RNase I digestions, although quantitatively the ribosome density was variable (Figure 4a). This was especially prominent within reads on 3′UTRs where MN produced ∼10× more footprints, but nevertheless locations of the RPFs were very similar (Figure 4b and Supplementary Figure S4c). The presence of RPFs far away from the coding sequences (e.g. >1.5 kb in YWHAZ/14-3-3z) and beyond multiple in frame and out of frame stop codons suggests that most of the 3′UTR reads do not derive from stop codon read-through (Figure 4b). The RPF counts on 3′UTRs for each gene correlated well between the digestion methods, especially in U2OS cells (*R*^2^ = 0.86, Figure 4c). The lower correlation in Kc167 (*R*^2^ = 0.41, Supplementary Figure S4d) is likely due to low 3′UTR read counts in RNase I sample resulting in high noise. The similar read locations argue that both digestion methods derive most of their 3′UTR RPFs from a common source.

Analysis of the RPF densities within relative rather than absolute positions along UTRs further support a common source for the 3′UTR reads as the 3′UTR density profiles are similar with both digestions (Figure 4d). In contrast, RNase I shows increased density in 5′UTR in the vicinity of start codon, consistent with known cycloheximide effects (12,37). The 3′UTR ribosome density continuously drops forming a ramp, arguing against post-lysis interactions and instead suggesting that these are post-termination ribosomes that fall off slowly (Figure 4d, Supplementary Figure S4e).

### Comparison of ribosome profiling conditions with MN digestion

According to the canonical view of translation ribosomes should not be present in the 3′UTRs. Therefore, to confirm our results, we varied the lysis and digestion conditions in U2OS cells using MN digestion to see if and how the presence of 3′UTR reads is dependent on the experimental conditions. The conditions we used before (control) replicated the previous observation of high abundance RPFs on 3′UTRs (Figure 5a and b, Supplementary Table S4). However, when using condition more similar to what others have used with MN (8,17), in other words reducing the salt condition or increasing the MN concentration, we observed a reduction in 3′UTR RPFs. This was independent on whether NaCl of KCl was used. The use of TritonX-100 as a detergent instead of NP40 and deoxycholate increased the RPFs in 3′UTRs. Correlations between the total RPF counts on cDNA matched with 3′UTR abundance (Supplementary Figure S5a). The amount of 5′UTR RPFs was not sensitive to the conditions tested. Together these data establish that ribosome profiling conditions can have a major impact on footprint quantities and can be modified to observe high abundance RPFs on the 3′UTRs.

We also tested if our FBS stimulation before sample collection affected results, but we did not observe any major changes on 3′UTR read abundance, although we observed ∼4× increase in serum responsive genes such as FOS and JUN (data not shown). In addition, 3′UTR reads were also observed in HeLa cells, although the levels were somewhat lower than in U2OS and Kc167 cells (Figure 5a and b).

### Affinity purification of ribosomes confirms pervasive interactions of 80S ribosomes with 3′UTR

As the extraction of ribosomes for ribosome profiling was done using sucrose cushion centrifugation, it is possible that the RPFs originate from non-ribosomal sources. To exclude this possibility we generated Kc167 cells stably expressing tagged ribosomal proteins RpS8, RpL13 or RpL22 and immunoprecipitated ribosomes under high salt conditions to minimize footprints originating from protection by non-ribosomal proteins. Analysis of these cell lines by western blotting indicated that the tagged proteins quantitatively accumulated in the crude ribosomal fraction indicating incorporation to ribosomes (Figure 5c). Ribosome profiling of the affinity purified ribosomes displayed similar RPF distribution on mRNAs as the samples purified with sucrose cushion (Figures 2a and 5d) with a high RPF content on the 3′UTRs (Figures 2c and 5f). The RPF counts per gene correlated well between the RpL13 and RpL22 samples (*R*^2^ = 0.91) (Supplementary Table S5). Similarly to the sucrose cushion purified samples, the affinity purified RPFs also displayed clear reduction in the mean RPF length outside CDS (Figure 5e), again suggesting a change in ribosome conformation or composition. It is also noteworthy that although we performed the immunoprecipitation of ribosomes overnight, the 3′UTR reads were not depleted. This does not reflect the translational status of the ribosomes as cycloheximide apparently also arrests non-translating ribosomes on long non-coding RNAs (6). Omitting cycloheximide in U2OS cells did not result in specific loss of 3′UTR reads (Supplementary Figure S5b). The affinity purified ribosome profiling results thus match the sucrose cushion based data, suggesting that most of the 3′UTR RPFs originate from interactions with 80S ribosomes. As we see similar RPF profile by immunoprecipitation of RpS13, which is part of the 40S subunit, these results are consistent with the observations in yeast that 3′UTR RPFs originate from 80S ribosomes (5).

It has been suggested that 3′UTR could associate with ribosomes on CDS (38). As these observations cannot exclude the possibility that the 3′UTR RPFs originate from mRNA associating with ribosome surface, we added excess RNA from mouse liver to the U2OS cell lysate before digestion to compete out possible non-specific ribosome binding to mRNA. Although the absolute RPF counts mapping to human cDNA dropped significantly as did the RPFs mapping to 3′UTRs, the relative read counts on 3′UTRs remained high (Supplementary Figure S5b). Although we could identify RPFs mapping exclusively to 5′UTR, 3′UTR and CDS of mouse transcripts, the low counts precluded reliable analysis of these, but this data nevertheless indicates that some of the ribosome footprints may originate from *in vitro* interactions (see also (39)). However, a recent analysis of post lysis ribosome interactions with mRNA indicated that most such interactions would occur with 5′UTR, not 3′UTR (37). In summary, while we cannot exclude the possibility that at least part of the observed 3′UTR RPFs originate from interactions with ribosomes outside of the mRNA channel, this data indicates that the 3′UTRs interact with 80S ribosomes extensively.

### 3′UTR ribosome occupancy is mainly dependent on relative 3′UTR length

**Figure 6.**
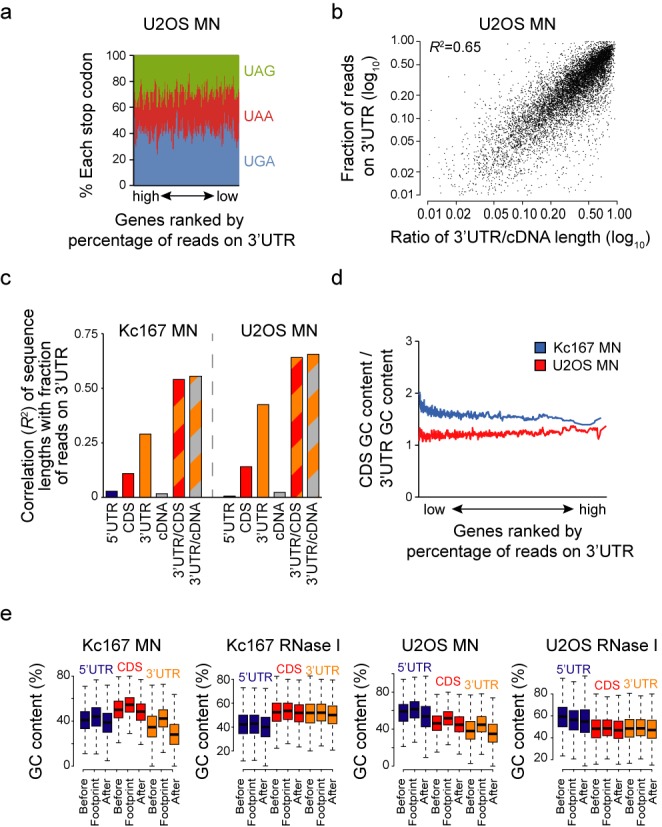
Relative mRNA region lengths largely define ribosome occupancy on 3′UTR. (**a**) Presence of 3′UTR RPFs does not depend on stop codon context. Genes in U2OS MN sample were ranked by the percentage of reads on 3′UTR and percentage of each stop codon in a 50 gene sliding window was plotted. (**b**) Correlation of relative 3′UTR length (as compared to CDS length) with fraction of 3′UTR reads on each gene. All genes with more than 100 RPFs in U2OS MN samples were included in analysis. Pearson correlation (*R*^2^) is indicated. (**c**) Pearson correlations (*R*^2^) of sequence lengths (5′UTR, CDS, 3′UTR and cDNA) and relative sequence lengths (3′UTR/CDS and 3′UTR/cDNA) with the fraction of 3′UTR reads on each gene. Of all sequence length features, 3′UTR length relative to cDNA length best correlates with 3′UTR read accumulation. (**d**) Differences in GC content on CDSs and 3′UTRs do not explain ribosome accumulation on 3′UTRs, as the relative GC content of 3′UTR does not increase with the relative amount of RPFs on 3′UTR. (**e**) Local ribosome position in 5′UTR, CDS and 3′UTR depends partly on GC content. Boxplots show GC content for each identified footprint and sequences immediately before and after footprint of similar length. RPFs from both Kc167 and U2OS cells digested with RNase I and MN were analysed and plotted as indicated. All before footprint, at footprint and after footprint differences are statistically highly significant (*P*< 2 × 10^−16^, ANOVA with Tukey's post hoc test). All data is derived from the MN and RNase I digested and sucrose cushion separated samples shown in Figure 2.

The mechanism by which ribosomes enter the 3′UTR in yeast could not be traced back to specific sequence elements (5). We set out to determine if such rules could be found in our dataset. We first analysed if the presence of 3′UTR reads would depend on stop codon. When all mRNAs were ranked by percentage of reads in their 3′UTR, no selectivity for any of the stop codons was observed using a 50-gene sliding window (Figure 6a and Supplementary Figure S6a). Thus, none of the stop codons is substantially more ‘leaky’. 3′UTR reads were not enriched at putative start and stop codons as would be expected for translating ribosomes or ribosomes attempting re-initiation (Figure 4b and Supplementary Figure S4c). No specific GO terms common for both Kc167 and U2OS cells were enriched in genes ranked by percentage of 3′UTR reads in MN samples (not shown).

We next analysed correlation between percentage of 3′UTR reads and sequence length features. A strong positive correlation was found with 3′UTR length, especially when normalizing to cDNA length (*R*^2^ = 0.65; Figure 6b and c). One potential explanation for this result is that MN digestion has a strong A/T bias and this would enrich 3′UTR reads as 3′UTRs are more A/T rich than CDSs (Supplementary Figure S6b and c). If so, the relative GC content between CDS and 3′UTR of each gene should predict the relative abundance of RPFs in the 3′UTR. However, this was not the case (Figure 6d). MN can also digest double stranded nucleic acid, making RNA secondary structures a less likely explanation for the 3′UTR RPFs. Thus, the relative ribosome content on 3′UTR largely, but not exclusively, depends on relative length of 3′UTR and is not due to nuclease bias.

To broaden the analysis, we compared the local GC content of the RPFs to the sequence immediately before and after the footprint. On both CDS and UTRs, the sequences on the footprints were more GC-rich than similar sized regions upstream or downstream, especially in the MN treated samples (Figure 6e). Similar results were observed in RNase I digested samples, although to a lesser extent as lower read counts on 3′UTRs are subject to the background noise. Footprint enrichment on GC-rich regions was also found in MN treated S2 cell data by Dunn *et al.* (Supplementary Figure S6d) and similar observations were made in a recent re-analysis of several yeast ribosome profiling datasets (40). However, unlike this analysis which focused on CDSs, our results suggest that ribosome presence on GC-rich regions is independent of translation elongation and peptide synthesis, as similar results are seen on CDSs and UTRs. The largest changes were observed in 3′UTR of MN digested Kc167 cells, where the change in GC content corresponds to the change of three A or T nucleotides to G or C nucleotides within the RPF, which cannot be explained by the A/T cutting bias of MN. These findings suggests that the GC content of the mRNA slows down ribosomes independently of translation, assuming that when the region is covered by ribosomes for a larger amount of time (slower translation), this results in higher read counts. The increased GC content observed in the RPFs also suggests that local mRNA folding downstream of the ribosome may be less critical in determining ribosome speed than previously suggested (41).

## DISCUSSION

We identify major differences in ribosome profiling data when different nucleases are used for digestion. The most prominent features are the enrichment of short genes in RNase I data and the high RPF density in 3′UTR in MN data. This suggests that RNase I and MN appear to identify different populations of ribosomes with different efficacies. Based on our results the quantitativeness of ribosome profiling should be evaluated with caution and different nuclease options and digestion conditions investigated. Overestimation of initiation by RNase I is indeed supported by the original ribosome profiling paper, where significantly improved correlations with proteome were found when ribosome densities were normalized to gene length (12). However, it is difficult to conclusively confirm this, as protein degradation rate is not taken into account in these analyses. The lesser enrichment of RPFs on start codons using MN suggests that RNase I recognizes the conformation of the ribosome on the start codon better than MN.

The other major difference between nucleases, the presence of abundant 3′UTR reads, was unexpected. We have identified conditions where MN does not over digest ribosomes and reveals high abundance of RPFs in 3′UTRs (Figures 3e and 5b). These results point out that ribosome profiling is more sensitive to sample preparation than previously appreciated. This data also suggests that specific aspects of translation can be highlighted with appropriate modifications to the method. The sensitivity to digestion conditions may explain why others have not observed the abundant 3′UTR ribosomes with MN digestion (17). Despite quantitative differences, 3′UTR RPF locations were very similar between the two digestion methods, indicating a common source for the footprints (Figure 4). The differences between nucleases were not due to nucleotide bias in digestion (Figure 6d), but we speculate that this is rather due to their physical size and/or extent of digestion (see high MN sample in Figure 5b). MN is substantially smaller than RNase I (17 versus 30 kDa, respectively) and this could affect its accessibility to the mRNA substrate. It is also known that binding of release factors induces conformational changes in ribosome (42), which could change the accessibility of nucleases to ribosomes. Further work is required to probe the conformation and composition of the ribosomes associated with 3′UTR.

We show that the 3′UTR reads are ribosome-dependent as affinity purification of ribosomes resulted in high 3′UTR RPF abundance (Figure 5d). These results, together with the mass spectrometry data and sequence motif analyses (Figure 3a and Supplementary Figure S3), indicate that most of the 3′UTR RPFs originate from 80S ribosomes rather than from other sources, such as RBPs.

Ribosomes have been previously observed on 3′UTRs. Stop codon read-through was previously identified in *Drosophila* (8) and ribosomes can also be observed on 3′UTRs *in vitro* (16) and in mutant yeast cells, where termination is reduced (5). We observe 3′UTR RPFs on thousands of genes (Supplementary Figure S4a and b). The high abundance of 3′UTR RPFs also prevents reliable identification of translating ribosomes from non-translating ones in our dataset based on the relative RPF counts before and after stop codons, as has been done to show that ribosomes present on long non-coding RNAs are not translating (6) (see Figure 4b). Note also that the 3′UTR RPFs are shorter than those on CDS (Figures 2b–d, f–h and 5e) and the RPFs on 3′UTRs did not accumulate on start or stop codons (Figure 4b), as one would expect in the case of translation reinitiation or read-through. It seems most likely that the 3′UTR associated ribosomes are not translating the 3′UTR consistent with previous mass spectrometry analyses, which have been able to identify only a handful of novel small peptides deriving from annotated non-coding regions (43,44).

There are two likely explanations for the RPFs in the 3′UTRs. The 3′UTR RPFs may represent post-termination ribosomes, which are largely digested when using RNase I. This is supported by the observation that 3′UTR ribosomes are more abundant near the stop codon, forming a ribosome ‘drop-off ramp’ (Figure 4d), rather than being found in the end of the 3′UTR, as in yeast (5). Another possible explanation for the 3′UTR footprints is that the 3′UTR sequences interact with ribosomes on the CDS, as has been shown with specific mRNAs (38). Such interactions could be lost under different conditions. Whereas Eldad *et al*. (38) observed a decrease in association of 3′UTRs with ribosomes in higher salt concentrations, we observe that the 3′UTR RPFs appear more abundant in high salt conditions (Figure 5b), arguing against non-mRNA channel interactions. Our data cannot differentiate between these two options where ribosome footprints form from mRNA tunnel or surface interactions. Whereas further work is required to resolve the nature of 3′UTR association with ribosomes, it remains similarly possible that some of the 5′UTR footprints in ribosome profiling datasets could be due to post-lysis interactions or could involve interactions with the surface of the ribosomes.

**Figure 7.**
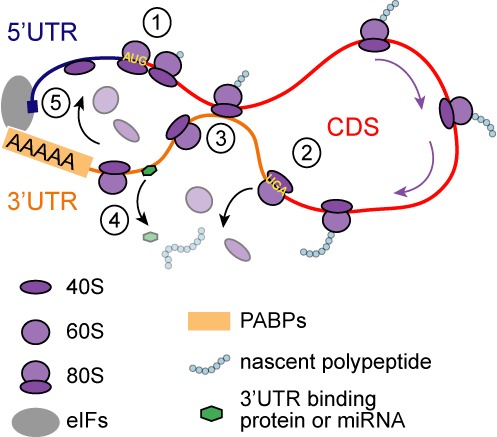
Potential explanations for ribosomes associated with 3′UTR. In canonical view of translation, ribosomes scan the 5′UTR, initiate translation from the AUG codon (**1**), and elongate along the CDS until the stop codon (UGA), where the produced polypeptide is released, ribosome disassembled and ribosome subunits detached from mRNA (**2**). The 3′UTR RPFs in our data can have two possible explanations. It is possible that 3′UTR sequences bind the ribosomes present in the CDS (**3**). This could affect ribosome migration and mRNA folding. Alternatively, although translation is typically terminated at the stop codon, 80S ribosomes could migrate to the 3′UTR (**4**). These 3′UTR ribosomes could detach or compete for the binding sites of other mRNA bound factors, which regulate translation. The movement of ribosomes to 3′UTR could also support ribosome recycling and initiation of a new round of translation when mRNA is in closed loop formation (**5**). PABP = PolyA-binding protein; eIFs = eukaryotic initiation factors.

The extensive interactions between 3′UTR sequences and ribosomes suggest a role in translational regulation. As it is commonly accepted that highly translated mRNA forms a loop through interactions between polyA binding proteins and initiation factors (3), it is possible that ribosomes may move through the 3′UTR to support recycling (45). If so, 3′UTR ribosome occupancy may vary largely between cell types, as does the importance of mRNA loop formation (3). Bringing terminated ribosomes closer to the polyA tail could enhance ribosome recycling and thus potentially translational efficiency. Consistent with this, the relative lengths of 3′UTRs explain most variation in ribosome occupancy of 3′UTRs (Figure 6c). In theory, the 3′UTR ribosomes may also be competing for binding sites or be involved in dislocation of other mRNA bound factors (41). Similarly, if the RPFs on 3′UTRs originate from interactions with ribosomes in the CDSs, it is possible that they affect ribosome elongation and mRNA folding. We have illustrated these concepts of translational regulation in Figure 7. Finally, if the ribosomes move along the 3′UTR, what is the energy source required for ribosome movement? The shape of the 3′UTR RPF density suggests that ribosomes fall off slowly after the stop codon. Thus, could it be that elongation factors, which normally provide energy for ribosome movement through guanosine-5'-triphosphate hydrolysis remain associated with the ribosomes after stop codon?

In conclusion, ribosome profiling is surprisingly sensitive to the digestion conditions and the results obtained with the method should be interpreted with care. We have identified conditions in which abundant 3′UTR RPFs can be observed. These RPFs originate from ribosomes which migrate through the stop codon very commonly or which are located on other part of mRNA, but still interact with 3′UTR sequences. As similar results are seen in both *Drosophila* and human cells, is seems possible that 3′UTRs may have a yet unknown role in translational regulation in metazoans.

## ACCESSION NUMBERS

The ArrayExpress accession number for the ribosome profiling data (fastq files) reported in this paper is E-MTAB-2421 and E-MTAB-3135.

## SUPPLEMENTARY DATA

Supplementary Data are available at NAR Online.

SUPPLEMENTARY DATA

SUPPLEMENTARY DATA
