# Supplementary material for: Modified ribosome profiling reveals high abundance of ribosome protected mRNA fragments derived from 3′ untranslated regions
Source: Nucleic Acids Res. 2014 Dec 29;43(2):1019–34. doi: 10.1093/nar/gku1310 (PMC4333376; doi:10.1093/nar/gku1310)
Supplement: SUPPLEMENTARY DATA [file supp_43_2_1019__index.html]

Modified ribosome profiling reveals high abundance of ribosome protected mRNA fragments derived from 3′ untranslated regions — SUPPLEMENTARY DATA 

# Modified ribosome profiling reveals high abundance of ribosome protected mRNA fragments derived from 3′ untranslated regions

## SUPPLEMENTARY DATA

**Files in this Data Supplement:**

- SUPPLEMENTARY DATA
- SUPPLEMENTARY DATA
